# Polypharmacy and Guideline-Directed Medical Therapy Initiation Among Adults Hospitalized With Heart Failure

**DOI:** 10.1016/j.jacadv.2024.101126

**Published:** 2024-08-05

**Authors:** Chukwuma Onyebeke, David Zhang, Mahad Musse, Ozan Unlu, Musarrat Nahid, Andrew P. Ambrosy, Emily B. Levitan, Monika M. Safford, Parag Goyal

**Affiliations:** a Department of Medicine, Weill Cornell Medicine, New York, New York, USA; b Program for the Care and Study of the Aging Heart, Weill Cornell Medicine, New York, New York, USA; c Division of Cardiovascular Medicine, Brigham and Women‘s Hospital, Mass General Brigham, Harvard Medical School, Boston, Massachusetts, USA; d Department of Cardiology, Kaiser Permanente San Francisco Medical Center, San Francisco, California, USA; e Department of Epidemiology, University of Alabama at Birmingham, Birmingham, Alabama, USA

**Keywords:** guideline-directed medical therapy, heart failure

## Abstract

**Background:**

Underprescribing of guideline-directed medical therapy (GDMT) for heart failure (HF) persists.

**Objectives:**

The purpose of this study was to assess polypharmacy as a barrier to GDMT.

**Methods:**

We examined participants hospitalized for HF with reduced ejection fraction and HF with mildly reduced ejection fraction between 2003 and 2017 from the Reasons for Geographic and Racial Differences in Stroke study. Participants were stratified by admission medication count—0 to 4, 5 to 9, and ≥10 medications. We examined GDMT use at admission, GDMT contraindications, and initiation of eligible indicated GDMT by medication count. We conducted a multivariable Poisson regression with robust standard errors to examine the association between medication count and GDMT initiation. GDMT included agents for HF with reduced ejection fraction/HF with mildly reduced ejection fraction, antiplatelet agents and statins for coronary artery disease, and anticoagulants for atrial fibrillation.

**Results:**

Among 545 participants with HF, 34% were not taking a beta-blocker, 39% were not taking an angiotensin-converting enzyme inhibitor/angiotensin receptor blocker/angiotensin receptor-neprilysin inhibitor, or hydralazine-isosorbide dinitrate, and 90% were not taking a mineralocorticoid receptor antagonist at admission; among participants with coronary artery disease, 36% were not taking an antiplatelet agent, and 38% were not taking a statin; and among participants with atrial fibrillation, 49% were not taking an anticoagulant. Polypharmacy was inversely associated with initiation of at least one indicated medication (5-9 medications: relative risk [RR]: 0.67; 95% CI: 0.56-0.82; *P* < 0.001; ≥10 medications: RR: 0.50; 95% CI: 0.39-0.64; *P* < 0.001) and initiation of at least half of indicated medications (5-9 medications: RR: 0.64; 95% CI: 0.51-0.81; *P* < 0.001; ≥10 medications: RR: 0.50; 95% CI: 0.38-0.67; *P* < 0.001).

**Conclusions:**

Polypharmacy is an important barrier to GDMT.

Neurohormonal antagonists, the cornerstone of guideline-directed medical therapy (GDMT) for heart failure with reduced ejection fraction (HFrEF), are persistently underprescribed despite their efficacy in reducing morbidity and mortality.^1^ In the Change the Management of Patients With Heart Failure (CHAMP-HF) registry of ambulatory HFrEF patients, 33% of eligible patients were not prescribed beta-blockers, 27% were not prescribed angiotensin-converting enzyme inhibitors/angiotensin II receptor blockers (ACEI/ARBs), and 67% were not prescribed mineralocorticoid receptor antagonists (MRAs).^2^ A study from the Get with the Guidelines-Heart Failure (GWTG-HF) registry revealed that among inpatient HFrEF patients being discharged, 5.4% were not prescribed beta-blockers, 7.3% were not prescribed ACEI/ARBs and 67.8% were not prescribed an MRAs.^3^ The harms of underprescribing GDMT are well-documented.^4^ Additionally, guideline-recommended medications for common cardiovascular comorbidities including coronary artery disease (CAD) and atrial fibrillation are also underprescribed. In prior studies of hospitalized patients with heart failure (HF) and CAD, 23% of patients were not prescribed aspirin, and 26% of patients were not prescribed statins at time of discharge.^5^^,^^6^ Among patients hospitalized for HF with atrial fibrillation, 31% were discharged without anticoagulation, according to a study from the GWTG-HF registry.^7^

The reasons for suboptimal rates of GDMT in HFrEF have been evaluated previously. Prescriber reasons for not prescribing GDMT include contraindications to neurohormonal blockade (renal dysfunction, hypotension, hyperkalemia, and bradycardia)^8^; concerns regarding patient nonadherence; and concerns about side effects and comorbidities.^9^^,^^10^ A survey of physicians treating patients with HFrEF indicated the possibility of an overlooked barrier—polypharmacy.^9^^,^^11^^,^^12^ Polypharmacy is defined as taking a high number of medications, with variable cutoffs described in the literature.^13^ Prior work has shown that polypharmacy is inversely associated with prescription of other guideline-concordant medications like statins and antihypertensives^14^^,^^15^; and recent secondary analysis of the GUIDE-IT (Guiding Evidence-Based Therapy Using Biomarker Intensified Treatment in Heart Failure) trial showed that polypharmacy was inversely associated with achieving optimal GDMT among ambulatory adults with HFrEF.^16^ However, no study to our knowledge has examined the association between polypharmacy and GDMT initiation among adults hospitalized for HF. Importantly, polypharmacy is nearly universal among older adults hospitalized for HF.^17^^,^^18^ To examine polypharmacy as a potential barrier to GDMT, we investigated whether polypharmacy was associated with in-hospital GDMT initiation among hospitalized patients with HFrEF and HF with mildly reduced ejection fraction (HFmrEF), which has been shown to benefit from similar agents as HFrEF.^19^, ^20^, ^21^

## Methods

### Study population

We performed a retrospective observational cohort study of participants with adjudicated HF admission between 2003 and 2017 from the REGARDS (Reasons for Geographic and Racial Differences in Stroke) study. We examined those with HFrEF or HFmrEF, defined as a left ventricular ejection fraction (LVEF) of ≤50% and/or a qualitative description of abnormal systolic function. We combined HFrEF and HFmrEF for the purposes of this analysis, given that pharmacologic recommendations for these HF subtypes are similar.^1^^,^^19^, ^20^, ^21^ We excluded participants who died during hospitalization, were referred to hospice, or lacked medication data at either admission or discharge. For those with multiple hospitalizations, we examined the first eligible hospitalization only.

### Data source

The REGARDS study is a national geographically diverse multicenter observational cohort of 30,219 community-dwelling White and Black men and women aged ≥45 years from 48 contiguous states of the United States and the District of Columbia. Participants were recruited from 2003 to 2007. Baseline participant data were collected from a 45-minute telephone call and an in-person visit with the collection of blood, urine samples, and physiologic measures. Participants received follow-up telephone calls every 6 months and reported hospitalizations due to cardiovascular disease. Two expert clinicians reviewed the inpatient records of reported hospitalizations due to a cardiovascular disease to determine if the reason for hospitalization was acute decompensated HF using a structured adjudication process based on symptoms, physical exam findings, laboratory results, imaging, and medical treatment. When the two expert clinicians disagreed, a committee made the final decision. All participants received information about the study and gave consent at the time of enrollment. The REGARDS study had approval from the institutional review boards governing research on human subjects at the participating centers. Additionally, Weill Cornell Medicine Institutional Review Board approved this ancillary study.

We used data from the REGARDS baseline assessment and abstracted medical charts from adjudicated HF hospitalizations. REGARDS baseline data included sex, self-identified race, highest educational attainment, household income, and geriatric conditions. Geriatric conditions included functional impairment (defined as physical component summary score from the Short Form 12 of <30, which has been used previously),^17^ cognitive impairment (defined as a six-item screener score of <5), history of falls (defined as having at least one self-reported fall), and hypoalbuminemia (albumin level ≤3.3 g/dL) as a marker of frailty.^17^^,^^22^ Data on admission, progress notes, consultation notes, discharge summaries, medication reconciliation reports, laboratory and diagnostic testing reports, and nursing notes were collected through chart review of each adjudicated HF hospitalization. We used data from a rigorous chart abstraction effort previously described^17^ to determine admission and discharge medications (specific medications and medication count), comorbid conditions (including CAD and atrial fibrillation), laboratory values at admission and discharge (including creatinine and potassium), vital signs at admission and discharge (including systolic blood pressure and heart rate), and intolerance or allergy to specific medications (including beta-blockers, angiotensin-converting enzyme inhibitor/angiotensin II receptor blocker/angiotensin receptor-neprilysin inhibitor (ACEI/ARB/ARNI), hydralazine-isosorbide dinitrate (HYD-ISD), MRAs, antiplatelets, statins).^18^

### Medication count

We classified medication count based on the number of standing medications present on admission. Participants were stratified into three groups: 0 to 4, 5 to 9 (commonly referred to as polypharmacy^17^), and ≥10 medications (commonly referred to as hyperpolypharmacy).^13^^,^^23^

### Indicated medications

We defined indicated medications as medications recommended for treatment based on clinical practice guidelines. For HFrEF/HFmrEF, we included beta-blockers, ACEI/ARB/ARNI, and MRAs.^1^ Given data to support HYD-ISD use in lieu of ACEI/ARB, we counted HYD/ISDN as an equivalent for ACEI/ARB/ARNI in Black participants.^1^^,^^24^ We did not include ivabradine in this study since this agent had not yet permeated the market as of 2017 (the last year of this cohort study), and we did not include sodium glucose cotransporter-2 inhibitors since the landmark studies of these agents were completed after the last year of this cohort study.^25^^,^^26^

For CAD, we included antiplatelet agents (aspirin, clopidogrel, ticagrelor, prasugrel, cilostazol, dipyridamole, and ticlopidine) and statins.^27^ For atrial fibrillation, we included anticoagulants (including coumadin, enoxaparin, apixaban, rivaroxaban, dabigatran, and edoxaban) among participants with high stroke risk (CHA_2_DS_2_-VASc score of at least 2), for which anticoagulation would be recommended.^28^

### Contraindications

Contraindications to beta-blockers included hypotension (defined as systolic blood pressure <100 mm Hg) or bradycardia (defined as heart failure <60) on admission or discharge and reported allergy or intolerance to beta blockers.^29^ Contraindications to ACEI/ARBs/ARNIs included acute kidney injury (defined as an increase in creatinine by 50% or 0.3 from admission to discharge),^30^ hyperkalemia (defined as K >5.5) at admission or discharge, hypotension at admission or discharge, reported allergy or intolerance to ACEI/ARB, and being on dialysis.^31^, ^32^, ^33^ Contraindications to HYD-ISD included hypotension at admission or discharge and concurrent phosphodiesterase inhibitor use at discharge.^24^^,^^34^ Contraindications to MRAs included creatinine ≥2.5 in men or creatinine ≥2 in women on admission or discharge,^35^ acute kidney injury, hyperkalemia at admission or discharge, and hypotension at admission or discharge.^36^ Contraindications to antiplatelet agents included history of peptic ulcer disease/gastrointestinal bleed, history of hemorrhagic stroke, thrombocytopenia documented in chart, and reported allergy or intolerance to aspirin.^37^ Contraindication to statins included an intolerance or allergy to statins. Contraindications to anticoagulation included peptic ulcer disease, gastrointestinal bleed, history of hemorrhagic stroke, thrombocytopenia, and a history of falls.^38^ Supplemental Table 1 summarizes these contraindications. Although many of the listed contraindications are not absolute, we selected these criteria conservatively to determine the presence of potential contraindications.

### Indicated medication eligible for initiation

We defined indicated medications eligible for initiation as indicated medications (guideline recommended for HFrEF/HFmrEF, CAD, and/or atrial fibrillation) that were not present on admission and had no contraindications.

### Statistical analysis

To describe patient demographics and clinical characteristics, we calculated descriptive statistics including frequencies, percentages, means, and standard deviations. We used chi-squared tests and Kurskal-Wallis tests/ANOVA for categorical and continuous variables, respectively, to identify significant differences (*P* < 0.05) across categories.

To describe medication patterns, we calculated the proportion of participants not taking indicated medications at admission, stratified by medication count. We also calculated the proportion of participants with contraindications to indicated medications, stratified by medication count.

We then calculated number of indicated medications eligible for initiation, the proportion of indicated medications initiated by hospital discharge, and the proportion of participants who had each indicated medication initiated by the time of hospital discharge, stratified by medication count. We also calculated the proportion of participants who had at least 1 eligible medication initiated by the time of hospital discharge, at least 50% of eligible medications initiated by the time of hospital discharge, and all eligible medications initiated by the time of hospital discharge, stratified by medication count.

Finally, we conducted a multivariable Poisson regression with robust standard errors to estimate the relative risk (RR) and 95% CIs for initiation of any eligible medication by the time of hospital discharge, initiation of at least 50% of eligible medications by the time of hospital discharge, and initiation of all eligible indicated medications by the time of hospital discharge after adjusting for covariates. Covariates were chosen according to the well-established Andersen model of health care utilization,^39^ which includes predisposing, enabling, and need factors as variables that influence health care utilization. We included the following variables: age, sex, race, highest educational attainment less than high school, household income <$20,000, functional impairment (defined as physical component summary score from the Short Form-12 of <30), cognitive impairment (defined as a six-item screener score of <5), history of falls (defined as having at least one self-reported fall), hypoalbuminemia (albumin level ≤3.3 g/dL as a marker of frailty^22^), and comorbidity counts. To account for missing covariates, we used multiple imputation via chained equations.^40^ All statistical analyses were performed in STATA version 17 (StataCorp LLC).

We also conducted multiple sensitivity analyses. First, we repeated all analyses for HFrEF (LVEF<40%) and HFmrEF (LVEF 40%-50%) separately, given differences in the strength of evidence and potential benefits of GDMT for HFrEF/HFmrEF.^1^ We also conducted a sensitivity analysis using a more conservative definition of hyperkalemia (K >5) for contraindications to ACEI/ARB/ARNI and MRAs.

## Results

### Study population

We examined 545 participants with HFrEF/HFmrEF admitted to 408 unique hospitals. The exclusion cascade is shown in Supplemental Figure 1. The mean age was 75.1 ± 9.5 years, 38% were female, and 45.9% were Black (Table 1). Approximately 23.7% had a yearly income of <$20,000 USD, and 17.6% had an educational attainment of less than a high school degree. The median number of comorbid conditions was 7.0 (IQR: 5.0-9.0). CAD was present among 82.0% of participants, and atrial fibrillation was present among 41.7% (among whom 99.6% had an elevated stroke risk based on a CHA_2_DS_2_-VASc score of ≥2). On admission, 71 participants (13.0%) were taking 0 to 4 medications, 217 (39.8%) were taking 5 to 9, and 257 (47.2%) were taking ≥10, in the Central Illustration. Table 1 shows patient characteristics according to medication count.

**Table 1:** Participant Characteristics Stratified by Medication Count

	All	Medication Count at Admission		
		0-4 Strata	5-9 Strata	>10 Strata
Age at admission, y	75.1 ± 9.5	74.9 ± 11.1	74.8 ± 9.8	75.3 ± 8.8
Female	207 (38.0)	26 (36.6)	82 (37.8)	99 (38.5)
Black	250 (45.9)	27 (38.0)	107 (49.3)	116 (45.1)
Income <$20,000	129 (23.7)	17 (23.9)	59 (27.2)	53 (20.6)
Highest educational attainment less than high school education	96 (17.6)	11 (15.5)	40 (18.4)	45 (17.5)
Comorbidity count	7.0 (5.0-9.0)	5.0 (3.0-7.0)	6.0 (5.0-8.0)	8.0 (7.0-10.0)
PCS <30	99 (19.2)	5 (7.4)	32 (15.5)	62 (25.6)
Cognitive impairment	45 (11.2)	5 (9.3)	20 (12.7)	20 (10.5)
Frailty	227 (52.9)	30 (58.8)	89 (53.6)	108 (50.9)
History of falls	99 (18.2)	12 (16.9)	30 (13.8)	57 (22.3)
Coronary artery disease	447 (82.0)	51 (71.8)	171 (78.8)	225 (87.5)
Atrial fibrillation	227 (41.7)	27 (38.0)	87 (40.1)	113 (44.0)
Beta blocker on admission	362 (66.4)	24 (33.8)	139 (64.1)	199 (77.4)
ACEI/ARB/ARNI on admission	315 (57.8)	20 (28.2)	127 (58.5)	168 (65.4)
HYD-ISD on admission	30 (5.5)	0 (0.0)	6 (2.8)	24 (9.3)
MRA on admission	55 (10.1)	0 (0.0)	20 (9.2)	35 (13.6)
Antiplatelet agent on admission	321 (58.9)	22 (31.0)	122 (56.2)	177 (68.9)
Statin on admission	309 (56.7)	18 (25.4)	113 (52.1)	178 (69.3)
Anticoagulant on admission	148 (27.2)	5 (7.0)	49 (22.6)	94 (36.6)

Values are mean ± SD, n (%), or median (IQR).

ACEI = angiotensin-converting enzyme inhibitor; ARB = angiotensin receptor blocker; ARNI = angiotensin receptor-neprilysin inhibitor; HYD-ISD = hydralazine-isosorbide dinitrate; MRA = mineralocorticoid receptor antagonist; PCS = physical component summary score from the Short Form-12.

**Central Illustration:**
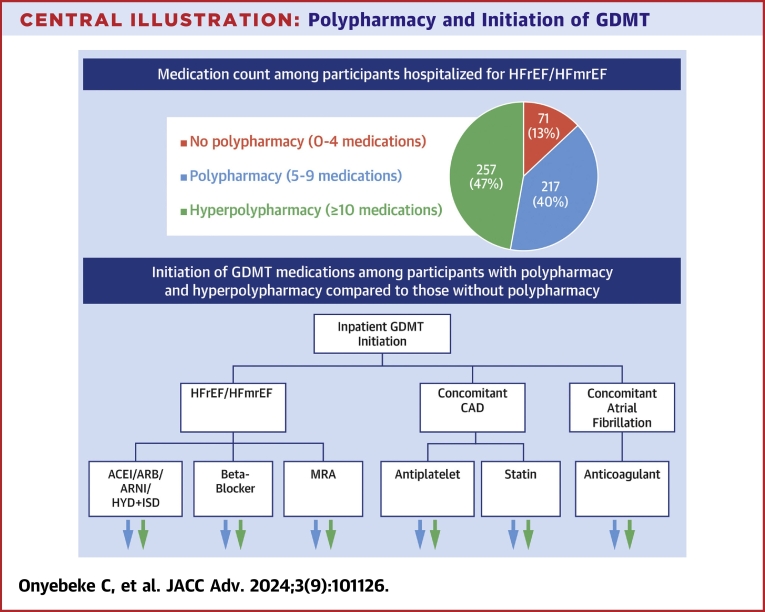
**Polypharmacy and Initiation of GDMT** The majority of participants admitted for HFrEF/HFmEF within the REGARDS cohort had either polypharmacy (5-9 medications) or hyperpolypharmacy (≥10 medications) at hospital admission. The percent of participants initiated on each eligible GDMT medication was lower among participants with polypharmacy and hyperpolypharmacy compared to those without polypharmacy. ACEI = angiotensin-converting enzyme inhibitor; ARB = angiotensin II receptor blocker; ARNI = angiotensin receptor-neprilysin inhibitor; CAD = coronary artery disease; GDMT = guideline-directed medical therapy; HFmrEF = heart failure with mildly reduced ejection fraction; HFrEF = heart failure with reduced ejection fraction; HYD-ISD = hydralazine-isosorbide dinitrate; REGARDS = Reasons for Geographic and Racial Disparities in Stroke.

### Prescribing patterns at hospital admission

Table 2 shows the proportion of participants who were not taking guideline-concordant medications at hospital admission. Regarding medications indicated for HFrEF/HFmrEF, 33.6% of participants were not taking a beta blocker, 39.5% were not taking an ACEI/ARB/ARNI or HYD-ISD, and 89.9% were not taking an MRA. Among participants with CAD, 36.0% were not taking an antiplatelet agent, and 38.5% were not taking a statin. Among participants with atrial fibrillation and a CHA_2_DS_2_-VASc score of ≥2, 49.6% were not taking an anticoagulant. With an increasing medication count at admission, the proportion of participants not taking each recommended medication class at the time of hospital admission decreased. These patterns were similar for HFrEF and HFmrEF (Supplemental Tables 2 and 3).

**Table 2:** Proportion of Participants Who Were Not Taking an Indicated Medication at Time of Admission, Stratified by Medication Count

	Total # Indicated for the Medication	Not Taking the Medication on Admission			
		All	Medication Count at Admission		
			0-4 Strata	5-9 Strata	>10 Strata
Beta blockers	545	183 (33.6)	47 (66.2)	78 (35.9)	58 (22.6)
ACEI/ARB/ARNI/HYD + ISD	545	215 (39.5)	51 (71.8)	85 (39.2)	79 (30.7)
MRA	545	490 (89.9)	71 (100)	197 (90.8)	222 (86.4)
Antiplatelet	447	161 (36.0)	33 (64.7)	65 (38.0)	63 (28.0)
Statin	447	172 (38.5)	37 (72.6)	75 (43.9)	60 (26.7)
Anticoagulant	226	112 (49.6)	22 (81.5)	49 (60.0)	41 (36.3)

Values are n (%).

ACEI = angiotensin-converting enzyme inhibitor; ARB = angiotensin receptor blocker; ARNI = angiotensin receptor-neprilysin inhibitor; HYD-ISD = hydralazine-isosorbide dinitrate; MRA = mineralocorticoid receptor antagonist.

### Patterns of contraindications to guideline concordant medications

Table 3 shows the prevalence of contraindications to GDMT among participants not taking an indicated medication on admission. Regarding medications indicated for HFrEF/HFmrEF, 19.1% of participants not taking beta-blocker had a contraindication, 45.6% of participants not taking ACEI/ARB/ARNI/HYD-ISD had a contraindication, and 41.0% of participants not taking MRA had a contraindication. For CAD, 10.7% of participants not taking an antiplatelet agent had a contraindication and 4.2% of participants not taking a statin had a contraindication. For atrial fibrillation, 7.3% of participants (with a CHA_2_DS_2_-VASc score of ≥2) not taking anticoagulation had a contraindication. These patterns were similar for HFrEF and HFmrEF (Supplemental Tables 4 and 5).

**Table 3:** Proportion of Participants Who Were Not Taking an Indicated Medication at Time of Admission Who Had a Contraindication, Stratified by Medication Count

	Contraindication				
	Total # Not on the Medication at Time of Admission	All	0-4 Strata	5-9 Strata	>10 Strata
Beta blocker	183	35 (19.1)	9 (19.2)	15 (19.2)	11 (19.0)
ACEI/ARB/ARNI/HYD + ISD	215	98 (45.6)	18 (35.3)	34 (40.0)	46 (58.2)
MRA	490	201 (41.0)	23 (32.4)	74 (37.6)	104 (46.9)
Antiplatelet	224	24 (10.7)	7 (14.3)	6 (6.3)	11 (13.8)
Statin	236	10 (4.2)	1 (1.9)	3 (2.9)	6 (7.6)
Anticoagulant	397	29 (7.3)	9 (13.6)	11 (6.6)	9 (5.5)

Values are n (%).

ACEI = angiotensin-converting enzyme inhibitor; ARB = angiotensin receptor blocker; ARNI = angiotensin receptor-neprilysin inhibitor; HYD-ISD = hydralazine-isosorbide dinitrate; MRA = mineralocorticoid receptor antagonist.

### Indicated medications eligible for initiation

The mean number of indicated medications eligible for initiation declined with increasing medication count—participants taking 0 to 4 medications at admission were eligible for initiation of a mean of 2.7 ± 1.4 medications, participants taking 5 to 9 medications at admission were eligible for initiation of a mean of 1.9 ± 1.1 medications, and participants taking ≥10 medications at admission were eligible for initiation of a mean of 1.3 ± 1.1 medications.

The proportion of indicated medications eligible for initiation that were initiated by hospital discharge decreased with an increasing medication count at admission. Among participants taking 0 to 4 medications at admission, 53.5% of indicated medications were initiated; among those taking 5 to 9 medications at admission, 34.7% of indicated medications were initiated; and among those taking ≥10 medications at admission, 28.1% of indicated medications were initiated.

**Figure 1:**
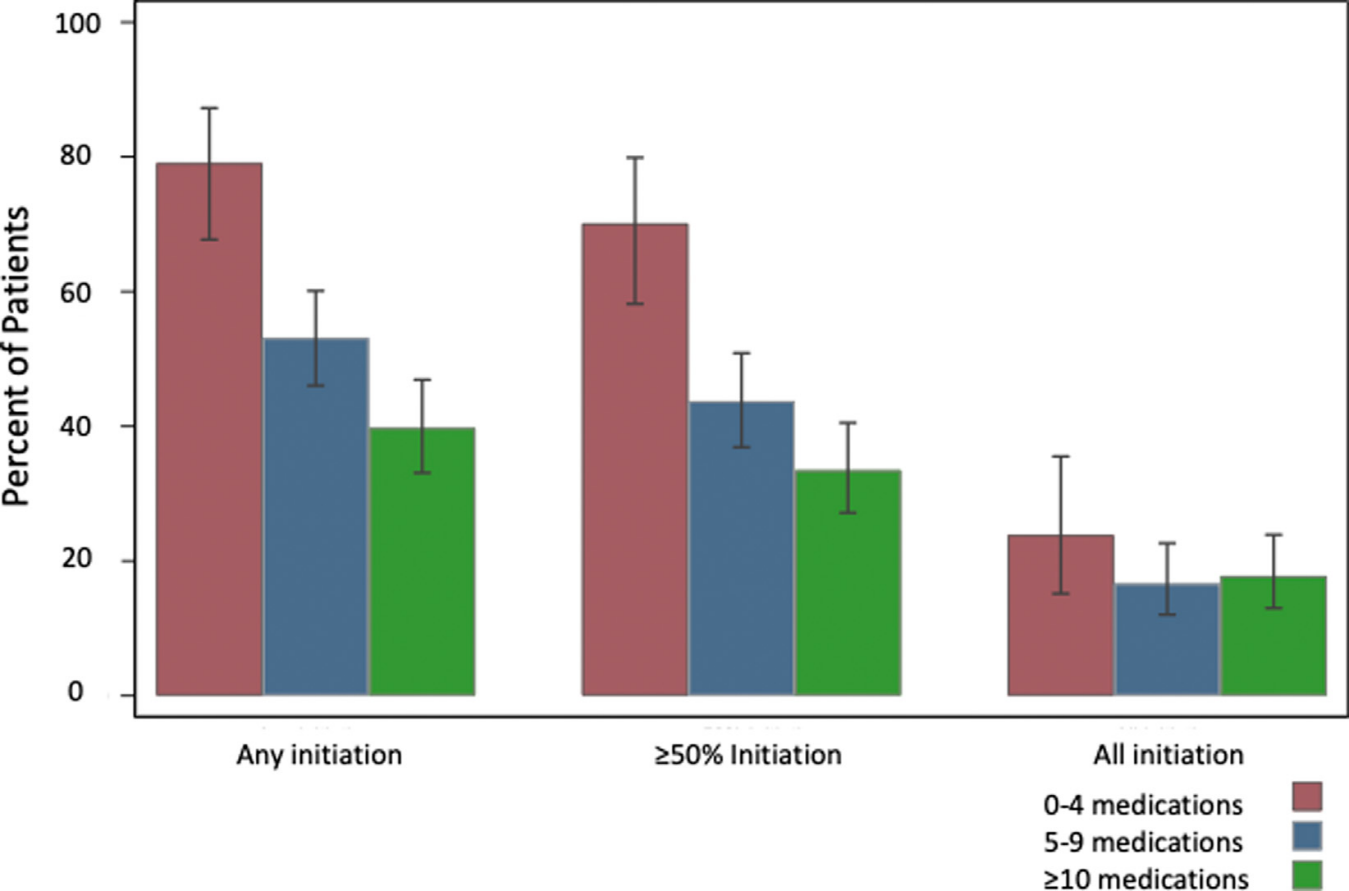
**Proportion of Participants Initiated on Any, at Least Half, and All Medications When Indicated and Eligible for Initiation, Stratified by Medication Count** The proportion of participants started on at least one, at least 50%, and all of indicated medications decreased with an increasing medication count at admission. Error bars indicate standard error.

Figure 1 shows the proportion of participants with any, at least one-half, and all initiation of indicated medications decreased with increasing medication count. Among participants taking 0 to 4 medications on admission, 79.1% were started on at least 1 indicated medication, 70.2% were started on at least half of indicated medications, and 23.9% were started on all indicated medications. Among participants taking 5 to 9 medications on admission, 53.1% were started on at least 1 indicated medication, 43.8% were started on at least half of indicated medications, and 16.7% were started on all eligible medications. Among participants taking ≥10 medications on admission, 39.8% were started on at least 1 indicated medication, 33.5% were started on at least half of indicated medications, and 17.8% were started on all indicated medications. These patterns were similar for HFrEF and HFmrEF (Supplemental Figures 2 and 3).

**Figure 2:**
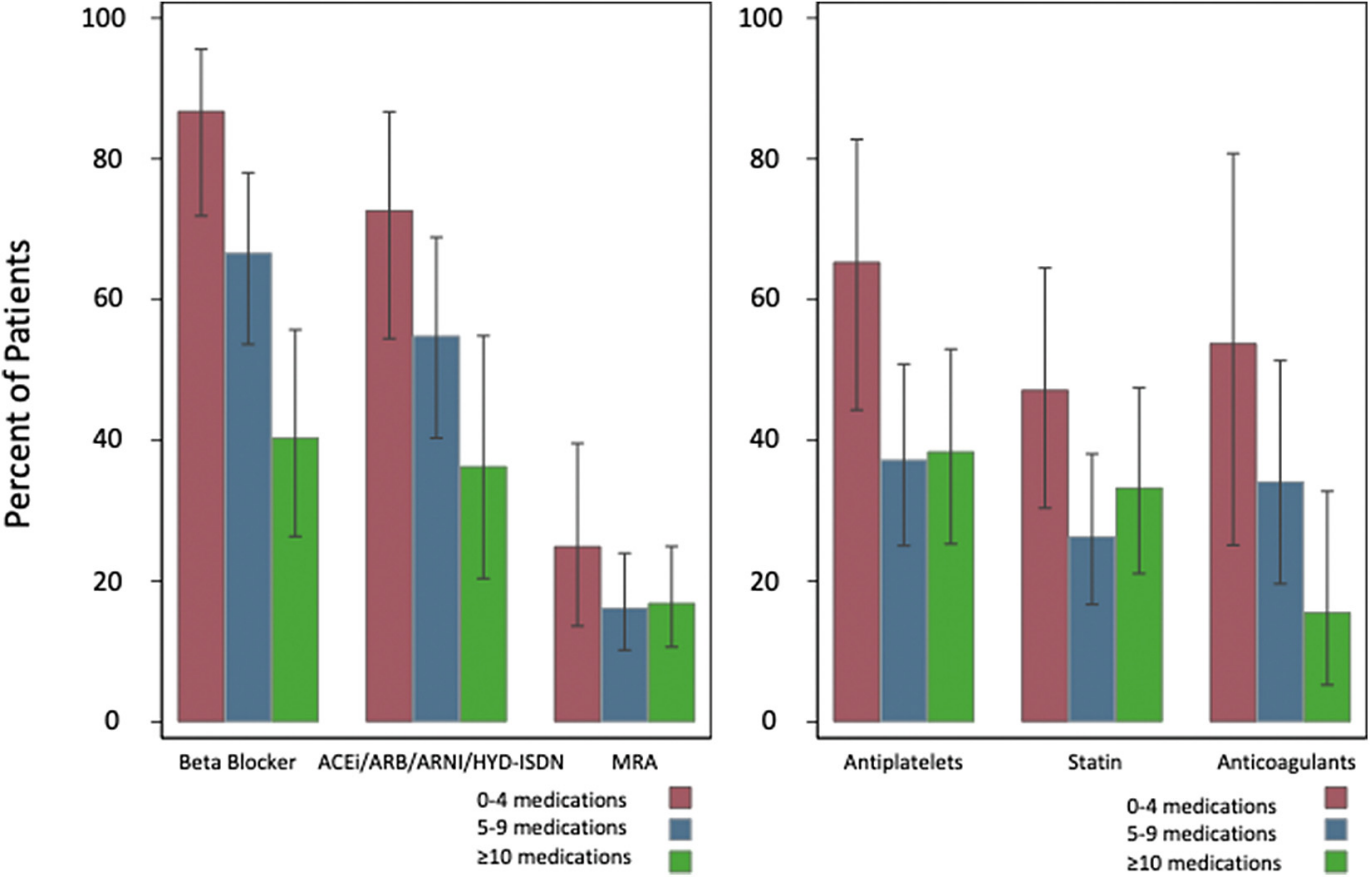
**Proportion of Participants Initiated on Each Medication When Indicated and Eligible for Initiation, Stratified by Medication Count** The proportion of participants with initiation of indicated beta-blockers, ACEI/ARB/ARNI/HYD-ISD, and anticoagulants decreased with an increasing medication count at admission. The proportion of participants with initiation of MRA, antiplatelet agents, and statins were highest for those who took 0 to 4 medications at admission and lower for those who took 5 to 9 and ≥10 medications at admission. Error bars indicate standard error. ACEI = angiotensin-converting enzyme inhibitor; ARB = angiotensin II receptor blocker; ARNI = angiotensin receptor-neprilysin inhibitor; HYD-ISD = hydralazine-isosorbide dinitrate; MRA = mineralocorticoid receptor antagonist.

Figure 2 shows the proportion of eligible participants who had a specified indicated agent initiated. The proportion of participants with initiation of indicated beta-blockers, ACEI/ARB/ARNI/HYD-ISD, and anticoagulants decreased with increasing medication count at admission. The proportions of participants with initiation of MRA, antiplatelet agents, and statins were lower for those who took 5 to 9 and ≥10 medications at admission compared to those who took 0 to 4 medications. These patterns were similar for HFrEF and HFmrEF (Supplemental Figures 4 and 5).

**Figure 3:**
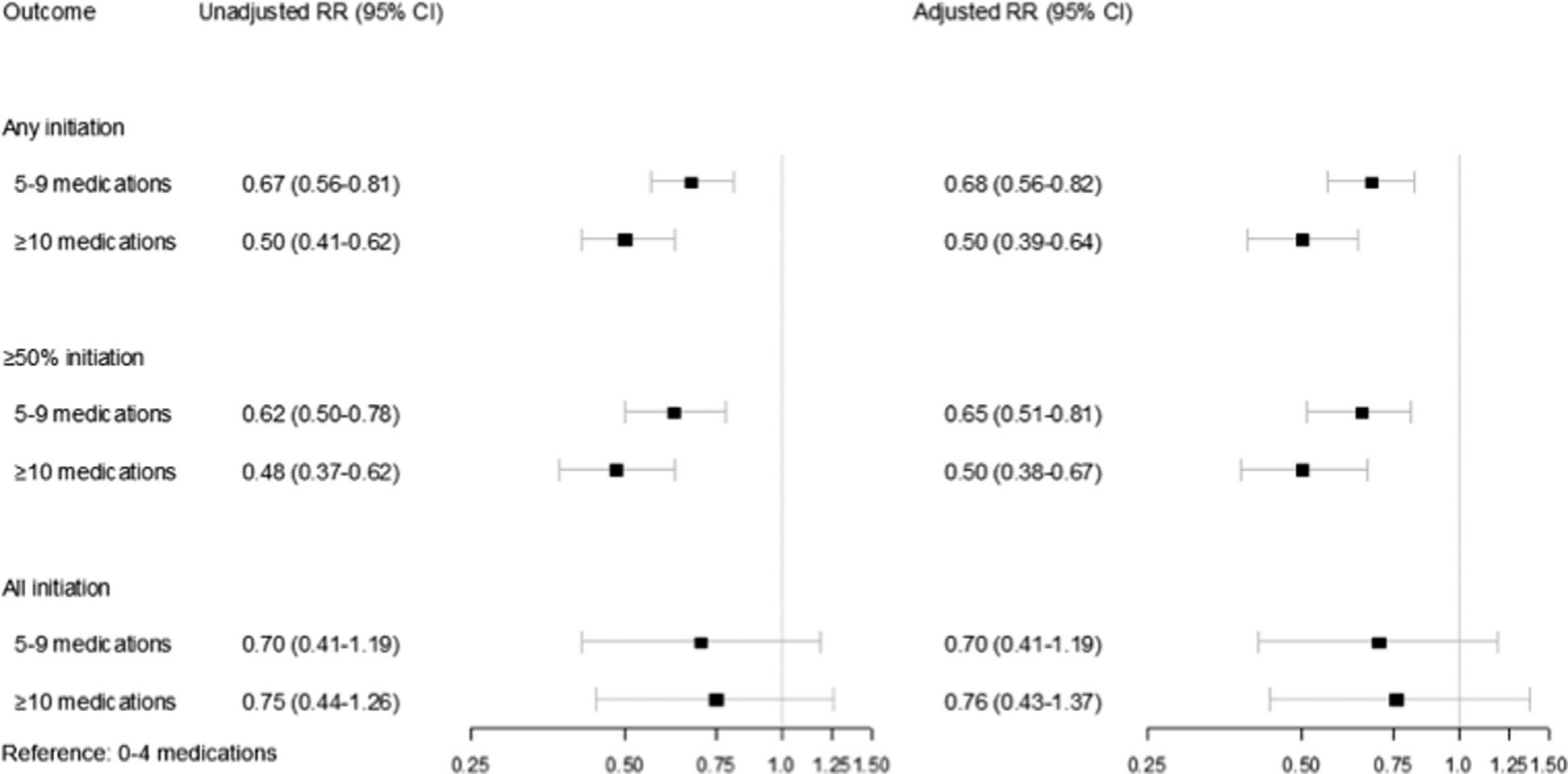
**Relative Risk of Initiation of Any, at Least Half, and All Medications According to Medication Burden** A multivariable-adjusted Poisson regression with robust standard errors estimating the relative risk (RR) and 95% CIs showed that taking 5 to 9 and ≥10 medications at the time of admission were inversely associated with initiation of at least one indicated medication and inversely associated with initiation of ≥50% of indicated medications. There was also a signal for an association between taking 5 to 9 and ≥10 medications at the time of admission and initiation of all indicated medications, but this did not meet statistical significance.

As shown in Figure 3, taking 5 to 9 and ≥10 medications on admission were inversely associated with initiation of at least one indicated medication compared to taking 0 to 4 medications at the time of admission (5-9 medications: RR: 0.68; 95% CI: 0.56-0.82; *P* < 0.001; ≥10 medications: RR: 0.50; 95% CI: 0.39-0.64; *P* < 0.001), and inversely associated with initiation of at least half of indicated medications (5-9 medications: RR: 0.65; 95% CI: 0.51-0.81; *P* < 0.001; ≥10 medications: RR: 0.50; 95% CI: 0.38-0.67; *P* < 0.001). The association between taking 5 to 9 and ≥10 medications on admission with the initiation of all indicated medications did not meet statistical significance (5-9 medications: RR: 0.70; 95% CI: 0.41-1.19; *P* = 0.18; ≥10 medications: RR: 0.76; 95% CI: 0.43-1.37; *P* = 0.36). These patterns were similar for HFrEF and HFmrEF (Supplemental Figures 6 and 7).

In a sensitivity analysis with hyperkalemia defined as K > s5, findings were similar (Supplemental Figures 8 to 10, Supplemental Table 6).

## Discussion

To our knowledge, this study from the geographically diverse REGARDS cohort is one of the first to describe the implications of polypharmacy on patterns of GDMT in adults with HFrEF/HFmrEF. We found that polypharmacy was inversely associated with GDMT initiation and that contraindications do not explain under-prescribing of GDMT. These findings have important implications given the prevalence of polypharmacy in older adults with HFrEF/HFmrEF.

Prior work has shown that polypharmacy is nearly universal in HFrEF/HFmrEF. In a study of older adults within the REGARDS cohort admitted with HF, 84% had polypharmacy (defined as at least 5 standing medications) and 42% had hyperpolypharmacy (defined as at least 10 standing medications).^18^ This is potentially problematic because polypharmacy is associated with an increased risk for adverse drug events, nonadherence, and treatment burden.^18^ Our findings identify another important negative effect of polypharmacy—GDMT underprescribing. Prior studies have shown that physicians report polypharmacy as a potential barrier to GDMT.^11^ Polypharmacy was reported as an important barrier to both beta-blocker initiation and uptitration in a survey among primary care providers.^11^ Our findings here quantify the negative impact of polypharmacy on GDMT prescribing—in particular, we found a graded reduction in the initiation of GDMT (when not present at admission and when no contraindications are present) with increasing medication count—those taking 5 to 9 medications (polypharmacy) were a third less likely to have an indicated medication initiated, and those taking at least 10 medications (hyperpolypharmacy) were nearly half as likely to have an indicated medication initiated compared to those taking <5 medications. These findings extend recent observations from secondary analysis of the Guiding Evidence-Based Therapy Using Biomarker Intensified Treatment in Heart Failure (GUIDE-IT) trial, which showed that polypharmacy was inversely associated with achieving optimal GDMT among ambulatory adults with HFrEF (mean age 61 years) over the span of 12 months.^16^ Together, these data support the need to develop strategies to combat polypharmacy as a barrier to GDMT. It is unknown whether involvement of a pharmacist in optimizing medication regimens could mitigate risks of polypharmacy, reduce unnecessary treatment burden, and/or address physician concerns about initiating medications for patients with an already high burden of medications.

Deprescribing is defined as the process of medication withdrawal under supervision to reduce medication-related adverse events^41^ and is a potentially helpful strategy for addressing polypharmacy. Discontinuing medications that exacerbate HF and/or potentially inappropriate medications according to the Beers criteria have been identified as potential strategies to improve the quality of medication prescribing in adults with HF.^42^^,^^43^ Replacing potassium with MRAs in those with hypokalemia may be another worthwhile strategy, given that this promotes GDMT without worsening polypharmacy.^18^ Whether these strategies focused on eliminating potential harmful agents can also facilitate increased use of GDMT is unknown and merits further investigation.

Importantly, we found that contraindications did not account for the majority of underprescribing. This is consistent with prior data on ambulatory patients with heart failure.^44^ Accordingly, some have suggested that underprescribing of GDMT is a consequence of clinical inertia,^45^ defined as delay or absence of initiation and escalation of indicated therapies.^45^ It is difficult to determine how much of GDMT underprescribing is from clinical inertia and how much is a consequence of a thoughtful balance of risks for harm and potential benefits. Future work focused on understanding physician perspectives on polypharmacy and medication risk is necessary. It is equally important to understand how physician (and patient) concerns about side effects and medication interactions are incorporated into decision-making when polypharmacy is present.

There are many reasons why a medication may not be prescribed, even when contraindications are absent. Patient preference is an important reason, though one would expect most patients to prefer initiation of therapies that can prevent morbidity and mortality. Another reason could be that prescribers believe that some patients will not live long enough to derive benefits from GDMT. Polypharmacy is a marker of comorbidity burden and is also associated with cognitive and functional impairment.^18^ Accordingly, some may perceive that patients with polypharmacy (especially more extreme forms) are less likely to benefit from GDMT. To help with such uncertainty, recent studies in HF have examined subgroups according to geriatric syndromes like frailty status.^46^ Incorporating geriatric syndromes such as these will continue to be critical to understand the benefits (and risks) of GDMT in real-world older adults who frequently contend with polypharmacy, frailty, and cognitive impairment, among other geriatric conditions.^24^^,^^47^

There are several strengths to this study. Geographic diversity was a major strength, as this cohort from REGARDS included participants admitted to 408 different hospitals across the 48 contiguous states of the United States and the District of Columbia. Another important strength was that the data was derived from chart level data, which permitted collection of detailed information on indications and contraindication, including data on intolerances/allergies as well as comorbid conditions.

### Study Limitations

There were also some important limitations. First, chart abstraction is an imperfect strategy to identify contraindications to medications, since conditions that could serve as a contraindication may not be clearly documented—it is possible that this study underestimated the prevalence of contraindications. Although we have used a broad list of conditions including relative contraindications that might prevent physicians from initiating GDMT in real practice, there may be other contraindications not clearly documented in the chart or not easily detected by chart review. In addition, there may have been discussions between the patient and physician regarding risks and benefits of GDMT that were not clearly documented, whereby the patient ultimately decided to defer initiation of a GDMT agent for various reasons. Second, we did not have data on prior attempts of GDMT and/or data from prior outpatient encounters that could have impacted GDMT initiation. Third, although we incorporated multiple covariates when examining the association between polypharmacy and GDMT initiation, following a well-accepted conceptual framework (the Andersen model), there is risk of unmeasured confounding. Fourth, there are important nuances to decision-making related to ACEI/ARB/ARNI vs HYD-ISD, specifically in Black persons that were beyond the scope of this study. Future work examining how polypharmacy and other factors affect the decision of prescribing ACEI/ARB/ARNI vs HYD-ISD is warranted. Fifth, we only examined underprescribing from the standpoint of initiation—whether polypharmacy impacts the likelihood of reaching maximal dosing is an important issue that also merits further investigation. Future work would also benefit from a larger sample size, as inferences regarding some of the medication patterns were limited by wide error bars. Finally, we included data as far back as 2003, though the majority of events occurred after 2010; since this time, the number of medications available to treat different conditions including HF has increased significantly. To build on this work, future work would benefit from examining medication patterns during the latest era of HF pharmacotherapy, which now notably includes sodium-glucose transport protein-2 inhibitors.

## Conclusions

Among older adults hospitalized for HFrEF/HFmrEF, polypharmacy and hyperpolypharmacy are inversely associated with initiation of GDMT. This suggests that polypharmacy is an important barrier to GDMT initiation among patient admitted for *HFrEF/HFmrEF*.

Perspectives **COMPETENCY IN MEDICAL KNOWLEDGE:** This study of 545 participants admitted for HFrEF/HFmrEF within the racially and geographically diverse REGARDS cohort reveals that polypharmacy is independently inversely associated with GDMT initiation, even when accounting for contraindications.

**TRANSLATIONAL OUTLOOK:** This data supports the need for developing strategies to combat polypharmacy. Such strategies may include greater involvement of pharmacists in optimizing medical regimens and a greater focus on deprescribing, especially among medications that exacerbate HF or are known to be harmful in elderly populations.

## Funding support and author disclosures

This research project is supported by cooperative agreement U01 NS041588, co-funded by the 10.13039/100000065National Institute of Neurological Disorders and Stroke (NINDS) and the 10.13039/100000049National Institute on Aging (NIA), the 10.13039/100000002National Institutes of Health, and the 10.13039/100000016Department of Health and Human Services. Additional funding was provided by 10.13039/100000050National Heart, Lung, and Blood Institute (NHLBI) grant R01HL080477 (Dr Safford) and 10.13039/100000049NIA grant R03AG056446 (Dr Goyal). Representatives from the 10.13039/100000050NHLBI did not have any role in the design and conduct of the study, the collection, management, analysis, and interpretation of the data, or the preparation or approval of the manuscript. Dr Goyal is supported by 10.13039/100000968American Heart Association grant 20CDA35310455, 10.13039/100000049National Institute on Aging grant K76AG064428; has received personal fees for medicolegal consulting related to heart failure; has received consulting fees from Sensorum Health; and has received honoraria from Akcea Therapeutics, Inc. Dr Ambrosy is supported by a Mentored Patient-Oriented Research Career Development Award (K23HL150159) through the 10.13039/100000050National Heart, Lung, and Blood Institute and has received relevant research support through grants to his institution from 10.13039/100000046Abbott, 10.13039/100014389Amarin Pharma, 10.13039/100006520Edwards Lifesciences, 10.13039/501100022336Esperion, Lexicon, and 10.13039/100008272Novartis. Dr Levitan has received research funding from 10.13039/100002429Amgen, Inc. All other authors have reported that they have no relationships relevant to the contents of this paper to disclose.

## References

[1] Heidenreich P.A., Bozkurt B., Aguilar D. (2022). 2022 AHA/ACC/HFSA guideline for the management of heart failure: a report of the American College of Cardiology/American heart association Joint committee on clinical practice guidelines. Circulation.

[2] Greene S.J., Butler J., Albert N.M. (2018). Medical therapy for heart failure with reduced ejection fraction: the CHAMP-HF registry. J Am Coll Cardiol.

[3] Krantz M.J., Ambardekar A.V., Kaltenbach L., Hernandez A.F., Heidenreich P.A., Fonarow G.C. (2011). Patterns and Predictors of evidence-based medication Continuation among hospitalized heart failure patients (from Get with the guidelines–heart failure). Am J Cardiol.

[4] Mebazaa A., Davison B., Chioncel O. (2022). Safety, tolerability and efficacy of up-titration of guideline-directed medical therapies for acute heart failure (STRONG-HF): a multinational, open-label, randomised, trial. Lancet.

[5] Bermingham M., Shanahan M.K., O’Connell E. (2014). Aspirin use in heart failure: is low-dose therapy associated with mortality and morbidity benefits in a large community population?. Circ Heart Fail.

[6] Ho P.M., Magid D.J., Shetterly S.M. (2008). Medication nonadherence is associated with a broad range of adverse outcomes in patients with coronary artery disease. Am Heart J.

[7] Eapen Z.J., Mi X., Qualls L.G. (2014). Adherence and persistence in the use of warfarin after hospital discharge among patients with heart failure and atrial fibrillation. J Card Fail.

[8] Gilstrap L.G., Stevenson L.W., Small R. (2018). Reasons for guideline nonadherence at heart failure discharge. J Am Heart Assoc.

[9] Erhardt L., Komajda M., Hobbs F.D.R., Soler-Soler J. (2008). Cardiologists’ awareness and perceptions of guidelines for chronic heart failure. The ADDress your Heart survey. Eur J Heart Fail.

[10] Steinman M.A., Dimaano L., Peterson C.A. (2013). Reasons for not prescribing guideline-recommended medications to adults with heart failure. Med Care.

[11] Levitan E.B., Van Dyke M.K., Loop M.S., O’Beirne R., Safford M.M. (2017). Barriers to beta-blocker Use and up-titration among patients with heart failure with reduced ejection fraction. Cardiovasc Drugs Ther.

[12] Steinman M.A., Patil S., Kamat P., Peterson C., Knight S.J. (2010). A taxonomy of reasons for not prescribing guideline-recommended medications for patients with heart failure. Am J Geriatr Pharmacother.

[13] Masnoon N., Shakib S., Kalisch-Ellett L., Caughey G.E. (2017). What is polypharmacy? A systematic review of definitions. BMC Geriatr.

[14] Kuijpers M.A.J., van Marum R.J., Egberts A.C.G., Jansen P.A.F. (2008). OLDY (OLd people Drugs & dYsregulations) Study Group. Relationship between polypharmacy and underprescribing. Br J Clin Pharmacol.

[15] Galvin R., Moriarty F., Cousins G. (2014). Prevalence of potentially inappropriate prescribing and prescribing omissions in older Irish adults: findings from the Irish LongituDinal Study on Ageing study (TILDA). Eur J Clin Pharmacol.

[16] Khan M.S., Singh S., Segar M.W. (2023). Polypharmacy and optimization of guideline-directed medical therapy in heart failure. JACC Heart Fail.

[17] Unlu O., Dharamdasani T., Archambault A. (2019). Polypharmacy increases in prevalence and Severity following a heart failure hospitalization. J Am Coll Cardiol.

[18] Unlu O., Levitan E.B., Reshetnyak E. (2020). Polypharmacy in older adults hospitalized for heart failure. Circ Heart Fail.

[19] Lund L.H., Claggett B., Liu J. (2018). Heart failure with mid-range ejection fraction in CHARM: characteristics, outcomes and effect of candesartan across the entire ejection fraction spectrum. Eur J Heart Fail.

[20] Enzan N., Matsushima S., Ide T. (2020). Spironolactone use is associated with improved outcomes in heart failure with mid-range ejection fraction. ESC Heart Fail.

[21] Wilcox J.E., Mann D.L. (2018). Beta-blockers for the treatment of heart failure with a mid-range ejection fraction: Deja-vu all over again?. Eur Heart J.

[22] Afilalo J., Alexander K.P., Mack M.J. (2014). Frailty assessment in the cardiovascular care of older adults. J Am Coll Cardiol.

[23] Kennel P.J., Kneifati-Hayek J., Bryan J. (2019). Prevalence and determinants of hyperpolypharmacy in adults with heart failure: an observational study from the national Health and Nutrition Examination survey (NHANES). BMC Cardiovasc Disord.

[24] Taylor A.L., Ziesche S., Yancy C. (2004). Combination of isosorbide dinitrate and hydralazine in blacks with heart failure. N Engl J Med.

[25] McMurray J.J.V., Solomon S.D., Inzucchi S.E. (2019). Dapagliflozin in patients with heart failure and reduced ejection fraction. N Engl J Med.

[26] Packer M., Anker S.D., Butler J. (2020). Cardiovascular and renal outcomes with Empagliflozin in heart failure. N Engl J Med.

[27] Fihn S.D., Gardin J.M., Abrams J. (2012). 2012 ACCF/AHA/ACP/AATS/PCNA/SCAI/STS guideline for the Diagnosis and management of patients with stable Ischemic heart disease. J Am Coll Cardiol.

[28] Calkins H., Chen L.Y., Joaquin Cigarroa FhrsE. (2019). 2019 AHA/ACC/HRS focused Update of the 2014 AHA/ACC/HRS guideline for the management of patients with atrial fibrillation: a report of the American College of Cardiology/American heart association Task Force on clinical practice guidelines and the heart Rhythm Society. J Am Coll Cardiol.

[29] Ko D.T., Hebert P.R., Coffey C.S. (2004). Adverse effects of β-blocker therapy for patients with heart failure: a Quantitative Overview of Randomized trials. Arch Intern Med.

[30] Thomas M.E., Blaine C., Dawnay A. (2015). The definition of acute kidney injury and its use in practice. Kidney Int.

[31] Bakris G.L., Weir M.R. (2000). Angiotensin-converting enzyme inhibitor-associated elevations in serum creatinine is this a cause for concern?. Arch Intern Med.

[32] Toto R.D., Mitchell H.C., Lee H.C., Milam C., Pettinger W.A. (2008). Reversible renal Insufficiency due to angiotensin converting enzyme inhibitors in Hypertensive Nephrosclerosis. Ann Intern Med.

[33] Kostis J.B., Shelton B., Gosselin G. (1996). Adverse effects of enalapril in the Studies of Left Ventricular Dysfunction (SOLVD). SOLVD Investigators. Am Heart J.

[34] Cheitlin M.D., Hutter A.M., Brindis R.G. (1999). Use of sildenafil (Viagra) in patients with cardiovascular disease. J Am Coll Cardiol.

[35] Ertram B., Itt P., Aiez F. (1999). The effect of spironolactone on morbidity and mortality in patients with severe heart failure. Randomized Aldactone Evaluation Study Investigators. N Engl J Med.

[36] Jonsson A., Norberg H., Bergdahl E., Lindmark K. (2018). Obstacles to mineralocorticoid receptor antagonists in a community-based heart failure population. Cardiovasc Ther.

[37] Kalyanasundaram A., Lincoff A.M. (2011). Managing adverse effects and drug–drug interactions of antiplatelet agents. Nat Rev Cardiol.

[38] Ageno W., Donadini M. (2018). Breadth of complications of long-term oral anticoagulant care. Hematology.

[39] Aday L.A., Andersen R. (1974). A framework for the study of access to medical care. Health Serv Res.

[40] Royston P, White IR. Multiple imputation by chained equations (MICE): implementation in stata. *J Stat Softw*, 45(4), 1-20.

[41] Krishnaswami A., Steinman M.A., Goyal P. (2019). Deprescribing in older adults with cardiovascular disease. J Am Coll Cardiol.

[42] Goyal P., Kneifati-Hayek J., Archambault A. (2020). Prescribing patterns of heart failure-Exacerbating medications following a heart failure hospitalization. JACC Heart Fail.

[43] Kennel P.J., Kneifati-Hayek J., Bryan J. (2018). Prevalence and determinants of hyperpolypharmacy in adults with heart failure. J Card Fail.

[44] Parajuli D.R., Shakib S., Eng-Frost J., McKinnon R.A., Caughey G.E., Whitehead D. (2021). Evaluation of the prescribing practice of guideline-directed medical therapy among ambulatory chronic heart failure patients. BMC Cardiovasc Disord.

[45] Greene S.J., Fonarow G.C. (2021). Clinical inertia and medical therapy for heart failure: the unintended harms of ‘first, do no harm.’. Eur J Heart Fail.

[46] Shahzeb Khan M., Segar M.W., Shariq Usman M. (2022). Frailty, guideline-directed medical therapy, and outcomes in HFrEF from the GUIDE-IT trial. JACC Heart Fail.

[47] Gorodeski E.Z., Goyal P., Hummel S.L. (2018). Domain management Approach to heart failure in the geriatric patient: present and future. J Am Coll Cardiol.

