# Supplementary material for: Polypharmacy and Guideline-Directed Medical Therapy Initiation Among Adults Hospitalized With Heart Failure
Source: JACC Adv. 2024 Aug 5;3(9):101126. doi: 10.1016/j.jacadv.2024.101126 (PMC11357976; doi:10.1016/j.jacadv.2024.101126)

Supplemental Table 1: Contraindications to medications

Medication	Contraindications
ACEi/ARB/ARNI	Acute kidney injury (defined as an increase in creatinine by 50% or 0.3 from admission to discharge), hyperkalemia (defined as K>5.5 mEq/L) at admission or discharge, hypotension at admission or discharge (defined as SBP<100 mmHg), reported allergy or intolerance to ACEi/ARB, and being on dialysis
HYD-ISD on admission	Hypotension at admission or discharge (defined as SBP<100 mmHg), and concurrent phosphodiesterase inhibitor use
MRA on admission	Cr≥2.5 mg/dL in men or Cr≥2 mg/dL in women on admission or discharge, acute kidney injury (defined as an increase in Cr by 50% or 0.3 mg/dL from admission to discharge), hyperkalemia (defined as K>5.5 mEq/L) at admission or discharge, and hypotension at admission or discharge (defined as SBP<100 mmHg)
Antiplatelet agent on admission	History of peptic ulcer disease/GI bleed, history of hemorrhagic stroke, thrombocytopenia documented in chart, and reported allergy or intolerance to aspirin
Statin on admission	Intolerance or allergy to statins
Anticoagulant on admission	Peptic ulcer disease, gastrointestinal bleed, history of hemorrhagic stroke, thrombocytopenia documented in chart, and a history of falls

Abbreviations: ACEi/ARB/ARNI: angiotensin-converting enzyme inhibitor/angiotensin receptor blocker/angiotensin receptor-neprilysin inhibitor; HYD-ISD: hydralazine-isosorbide dinitrate; MRA: mineralocorticoid receptor antagonist

Supplemental Table 2: Proportion of participants with HFrEF who were not taking an indicated medication at time of admission, stratified by medication count

Medication	***Total # indicated for the medication***	***Not taking the medication on admission***			
		All	*Medication count at admission*		
			0-4 strata	5-9 strata	≥10 strata
Beta blocker (n, %)	374	131 (35.0)	40 (71.4)	54 (36.2)	37 (21.9)
ACEi/ARB/ARNI/HYD+ISD (n, %)	374	142 (38.0)	40 (71.4)	57 (38.3)	45 (26.6)
MRA	374	334 (89.3)	56 (100)	135 (90.6)	143 (84.6)

Abbreviations: ACEi/ARB/ARNI: angiotensin-converting enzyme inhibitor/angiotensin receptor blocker/angiotensin receptor-neprilysin inhibitor; HYD-ISD: hydralazine-isosorbide dinitrate; MRA: mineralocorticoid receptor antagonist

Supplemental Table 3: Proportion of participants with HFmrEF who were not taking an indicated medication at time of admission, stratified by medication count

Medication	***Total # indicated for the medication***	***Not taking the medication on admission***			
		All	*Medication count at admission*		
			0-4 strata	5-9 strata	≥10 strata
Beta blocker (n, %)	158	47 (29.8)	6 (42.9)	21 (32.8)	20 (25)
ACEi/ARB/ARNI/HYD+ISD (n, %)	158	68 (43.0)	10 (71.4)	27 (42.2)	31 (38.8)
MRA	158	145 (91.8)	14 (100)	58 (90.6)	73 (91.2)

Abbreviations: ACEi/ARB/ARNI: angiotensin-converting enzyme inhibitor/angiotensin receptor blocker/angiotensin receptor-neprilysin inhibitor; HYD-ISD: hydralazine-isosorbide dinitrate; MRA: mineralocorticoid receptor antagonist

Supplemental Table 4: Proportion of participants with HFrEF who were not taking an indicated medication at time of admission who had a contraindication, stratified by medication count

		Contraindication			
Medication	Total # not on the medication at time of admission	All	0-4 strata	5-9 strata	≥10 strata
Beta blocker (n, %)	131	23 (17.6)	8 (20)	8 (14.8)	7 (18.9)
ACEi/ARB/ARNI/HYD+ISD* (n, %)	142	65 (45.8)	15 (37.5)	23 (40.4)	27 (60)
MRA (n, %)	334	128 (38.3)	19 (33.9)	46 (34.1)	63 (44.1)

Abbreviations: ACEi/ARB/ARNI: angiotensin-converting enzyme inhibitor/angiotensin receptor blocker/angiotensin receptor-neprilysin inhibitor; HYD-ISD: hydralazine-isosorbide dinitrate; MRA: mineralocorticoid receptor antagonist

Supplemental Table 5: Proportion of participants with HFmrEF who were not taking an indicated medication at time of admission who had a contraindication, stratified by medication count

		Contraindication			
Medication	Total # not on the medication at time of admission	All	0-4 strata	5-9 strata	≥10 strata
Beta blocker (n, %)	47	10 (21.3)	1 (16.7)	5 (23.8)	4 (20)
ACEi/ARB/ARNI/HYD+ISD* (n, %)	68	31 (45.6)	3 (30)	10 (37.0)	18 (58.1)
MRA (n, %)	145	66 (45.5)	4 (28.6)	24 (41.4)	38 (52.1)

Abbreviations: ACEi/ARB/ARNI: angiotensin-converting enzyme inhibitor/angiotensin receptor blocker/angiotensin receptor-neprilysin inhibitor; HYD-ISD: hydralazine-isosorbide dinitrate; MRA: mineralocorticoid receptor antagonist

Supplemental Table 6: Proportion of participants who were not taking an indicated medication at time of admission who had a contraindication (based on hyperkalemia defined as K>5), stratified by medication count

		Contraindication			
Medication	Total # not on the medication at time of admission	All	0-4 strata	5-9 strata	$\geq$10 strata
ACEi/ARB/ARNI/HYD+ISD* (n, %)	215	107 (49.8)	21 (41.2)	37 (43.5)	49 (62.0)
MRA (n, %)	490	217 (44.3)	26 (36.6)	81 (41.1)	110 (49.6)

Abbreviations: ACEi/ARB/ARNI: angiotensin-converting enzyme inhibitor/angiotensin receptor blocker/angiotensin receptor-neprilysin inhibitor; HYD-ISD: hydralazine-isosorbide dinitrate; MRA: mineralocorticoid receptor antagonist

Supplemental Figure 1: Exclusion **Cascade**

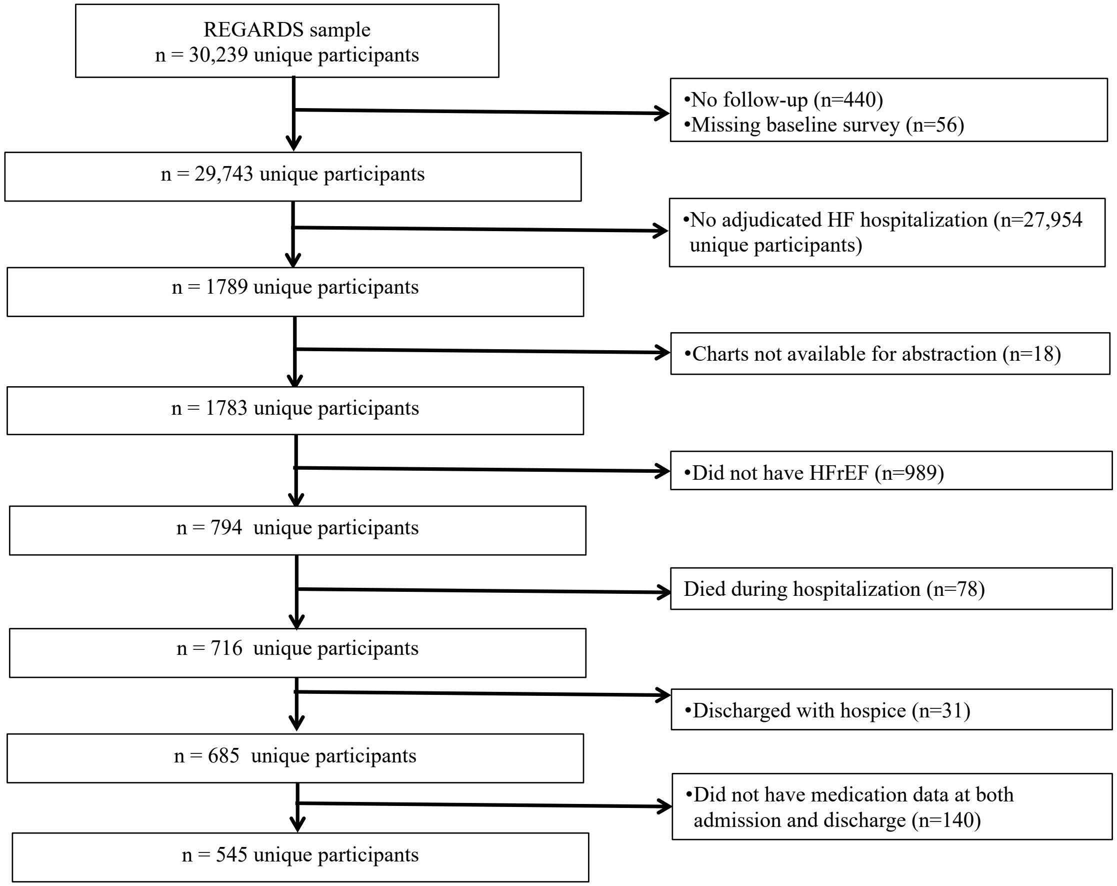

Supplemental Figure 2: Proportion of HFrEF participants initiated on any, at least half, and all medications when indicated and eligible for initiation, stratified by medication count

Supplemental Figure 2: Proportion of HFrEF participants initiated on any, at least half, and all medications when indicated and eligible for initiation, stratified by medication count

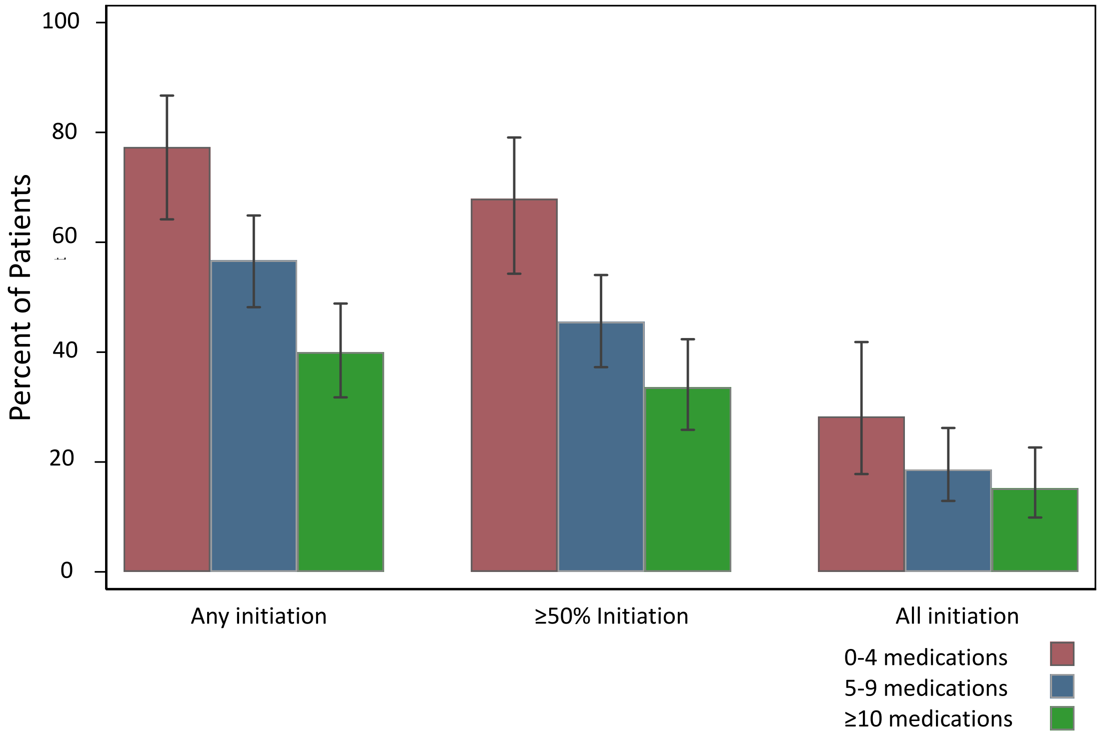

Supplemental Figure 3: Proportion of HFmrEF participants initiated on any, at least half, and all medications when indicated and eligible for initiation, stratified by medication count

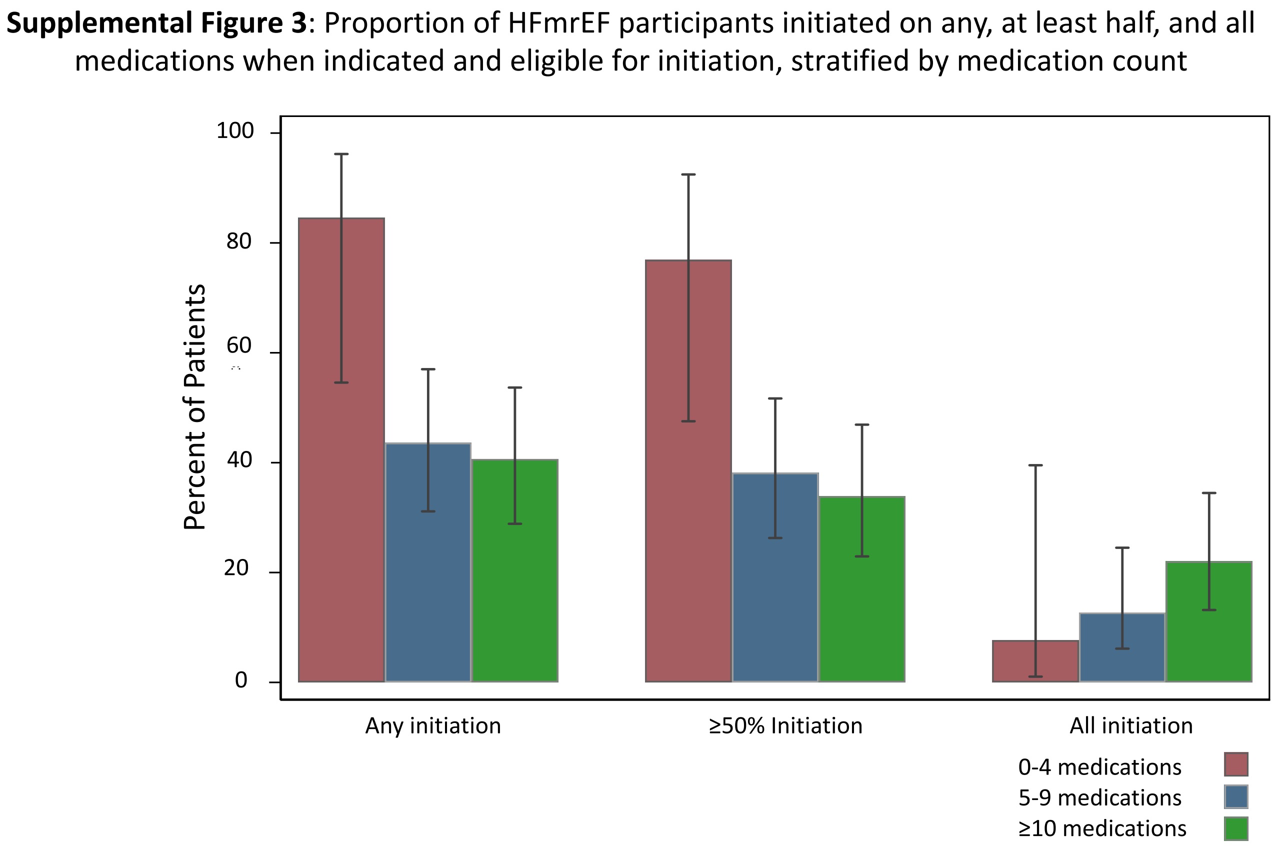

Supplemental Figure 4: Proportion of HFrEF participants initiated on each medication when indicated and eligible for initiation, stratified by medication count

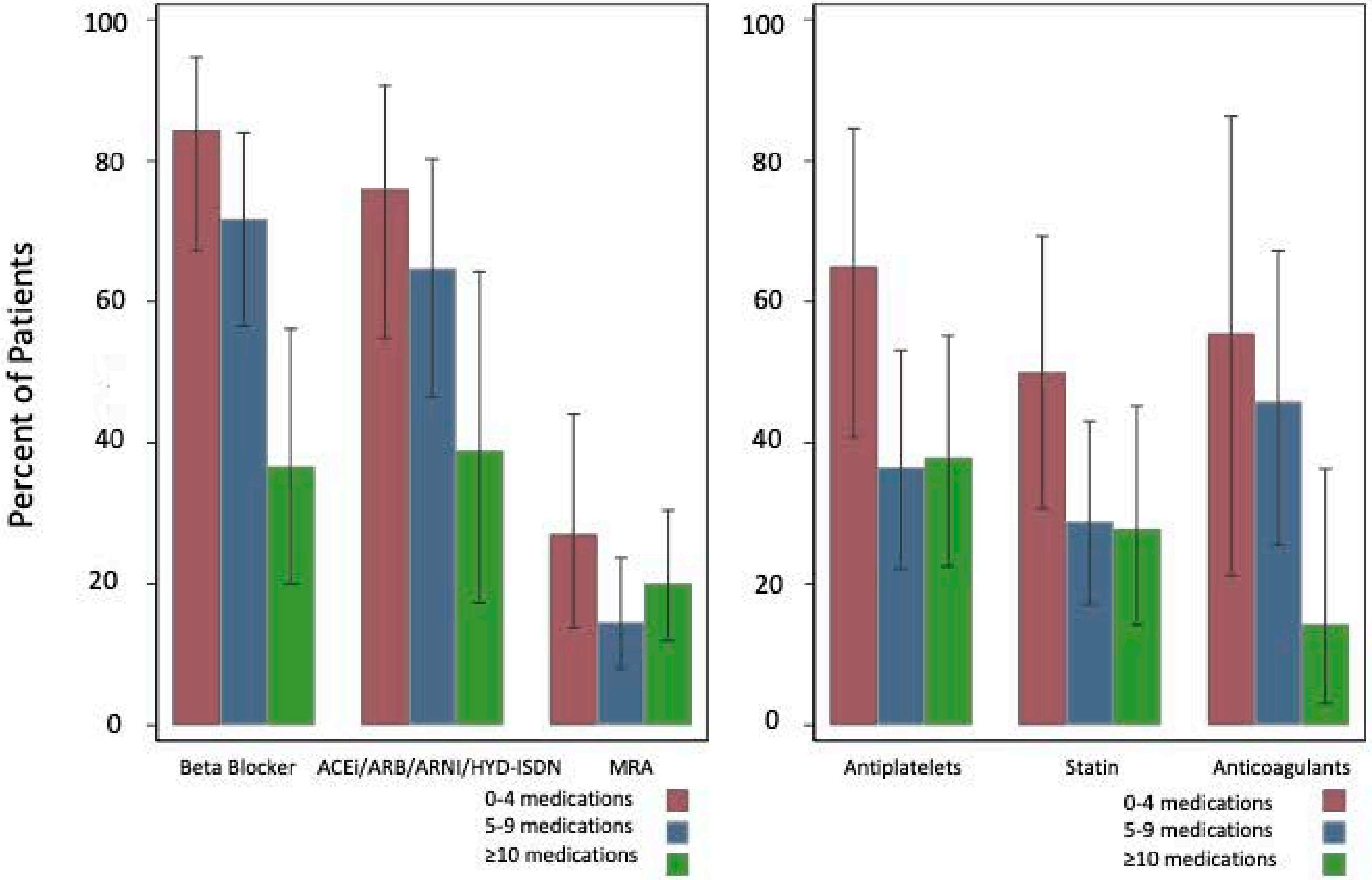

Supplemental Figure 5: Proportion of HFmrEF participants initiated on each medication when indicated and eligible for initiation, stratified by medication count

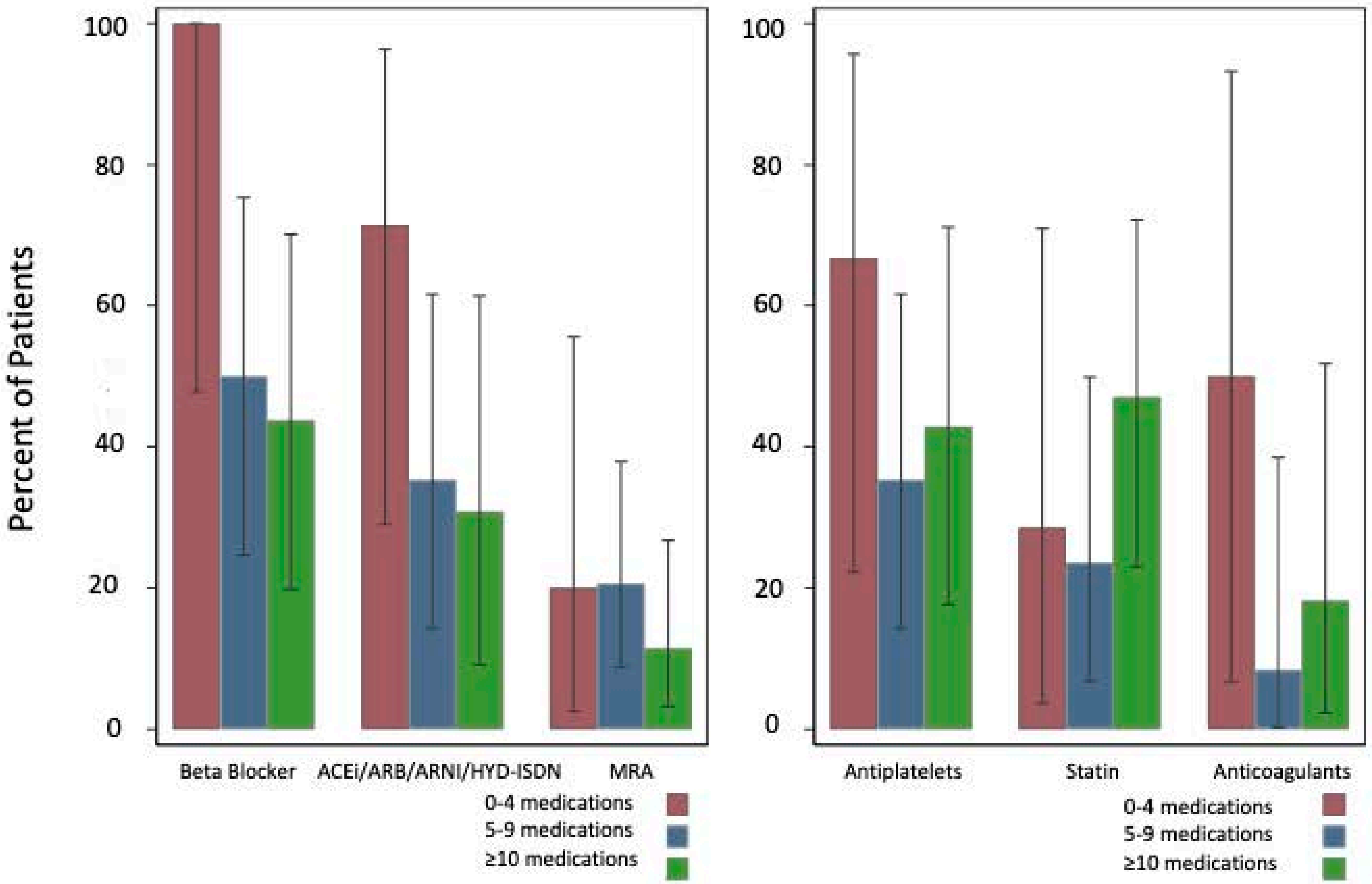

Supplemental Figure 6: Relative risk of initiation of any, at least half, and all medications according to medication burden among patients with HFrEF

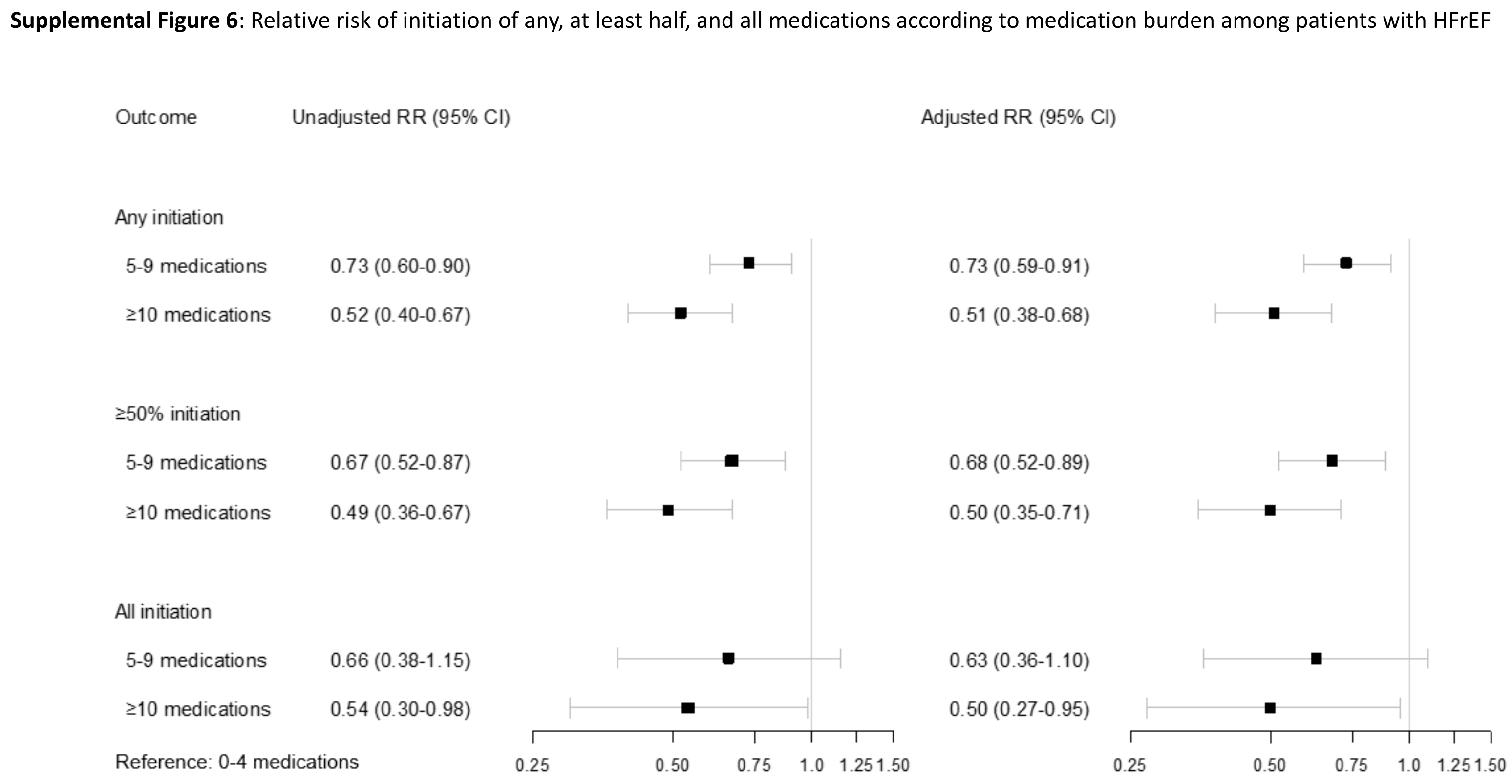

Supplemental Figure 7: Relative risk of initiation of any, at least half, and all medications according to medication burden among patients with HFmrEF

Supplemental Figure 7: Relative risk of initiation of any, at least half, and all medications according to medication burden among patients with HFmrEF

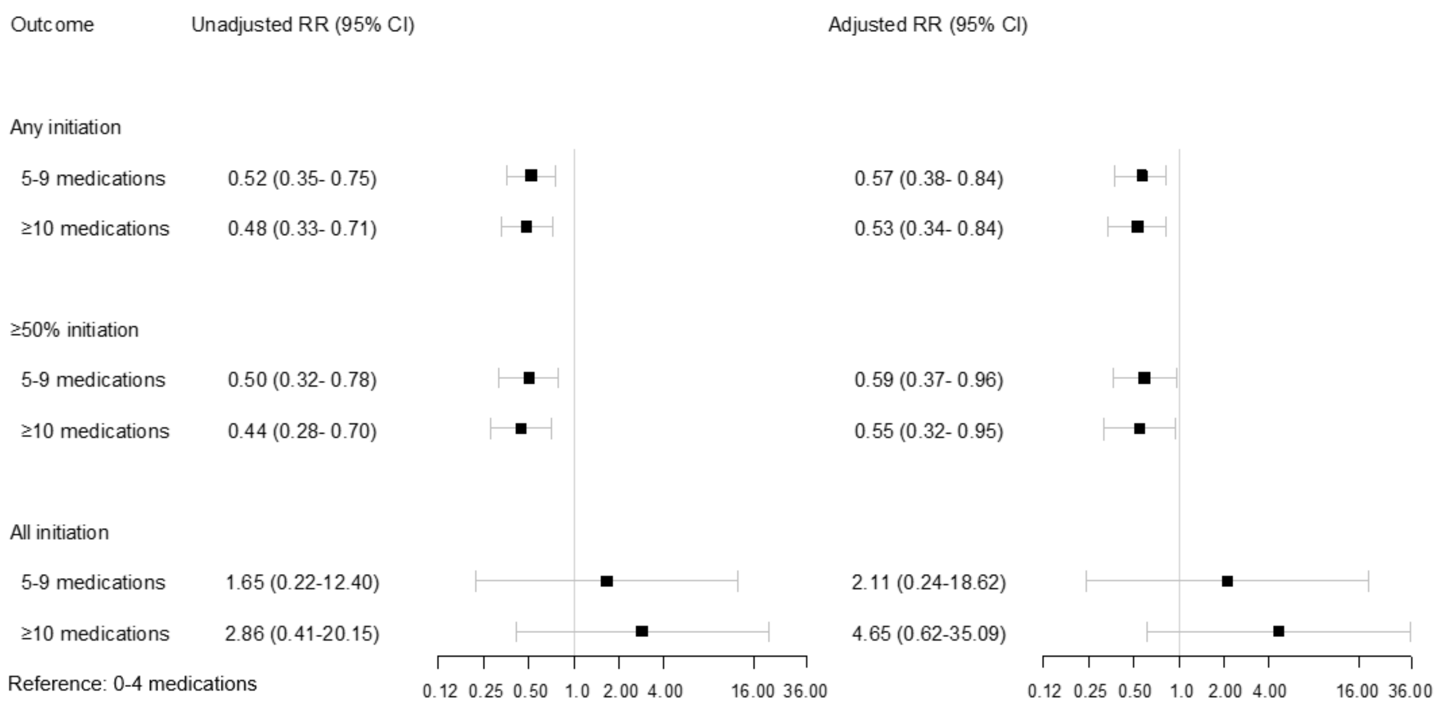

Supplemental Figure 8: Proportion of participants initiated on any, at least half, and all medications when indicated and eligible for initiation, stratified by medication count (based on hyperkalemia defined as K>5)

Supplemental Figure 8: Proportion of participants initiated on any, at least half, and all medications when indicated and eligible for initiation, stratified by medication count (based on hyperkalemia defined as K>5)

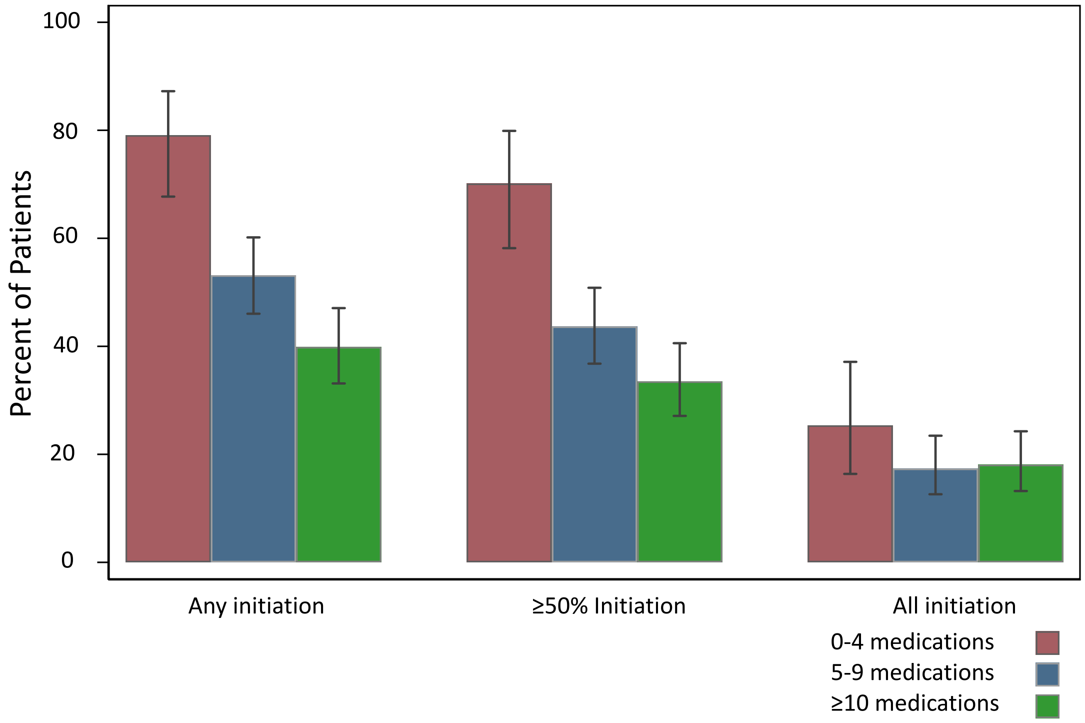

Supplemental Figure 9: Proportion of participants initiated on each medication when indicated and eligible for initiation, stratified by medication count (based on hyperkalemia defined as K>5)

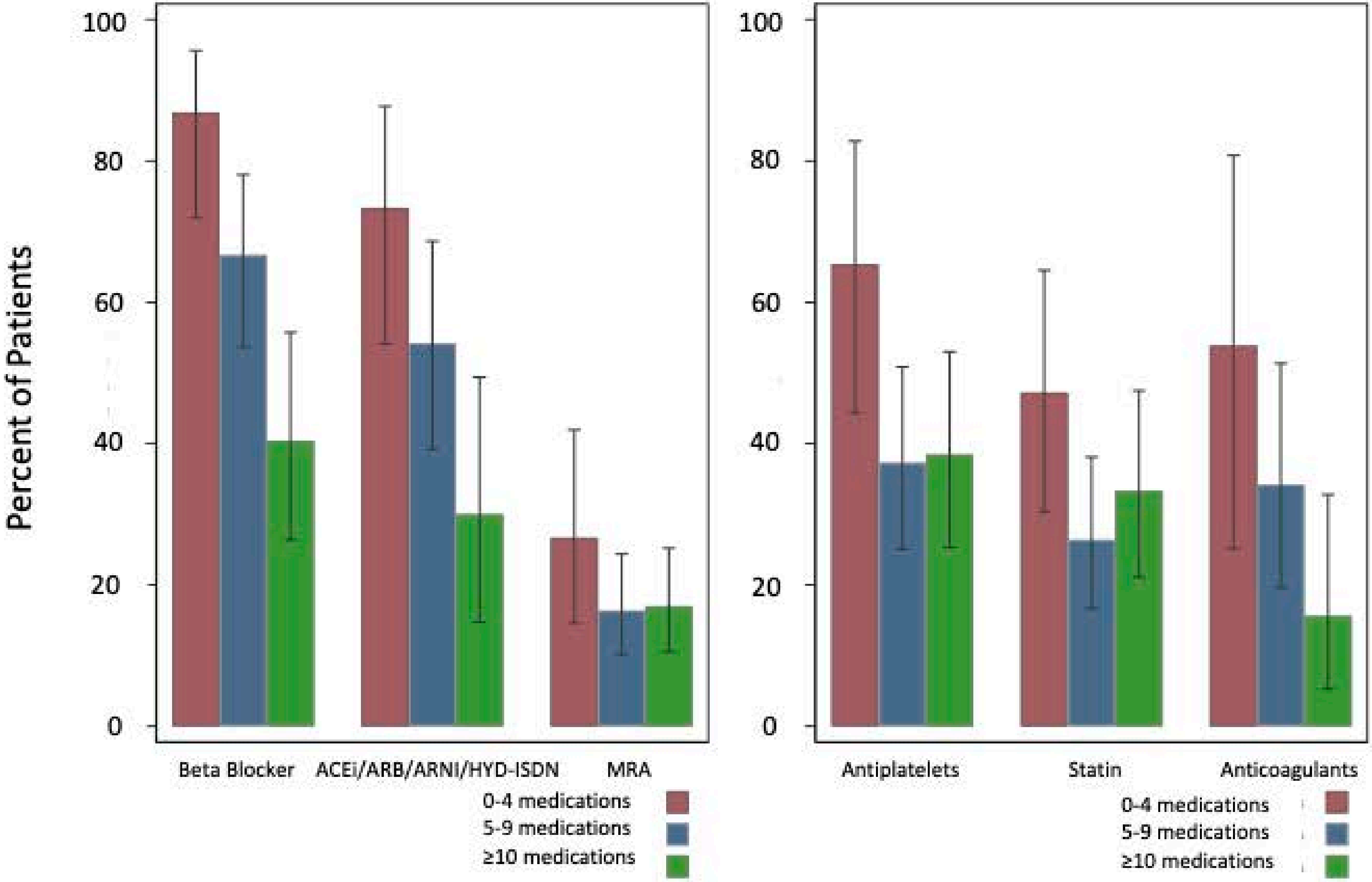

Supplemental Figure 10: Relative risk of initiation of any, at least half, and all medications according to medication count (based on hyperkalemia defined as K>5)

Supplemental Figure 10: Relative risk of initiation of any, at least half, and all medications according to medication burden (based on hyperkalemia defined as K>5)

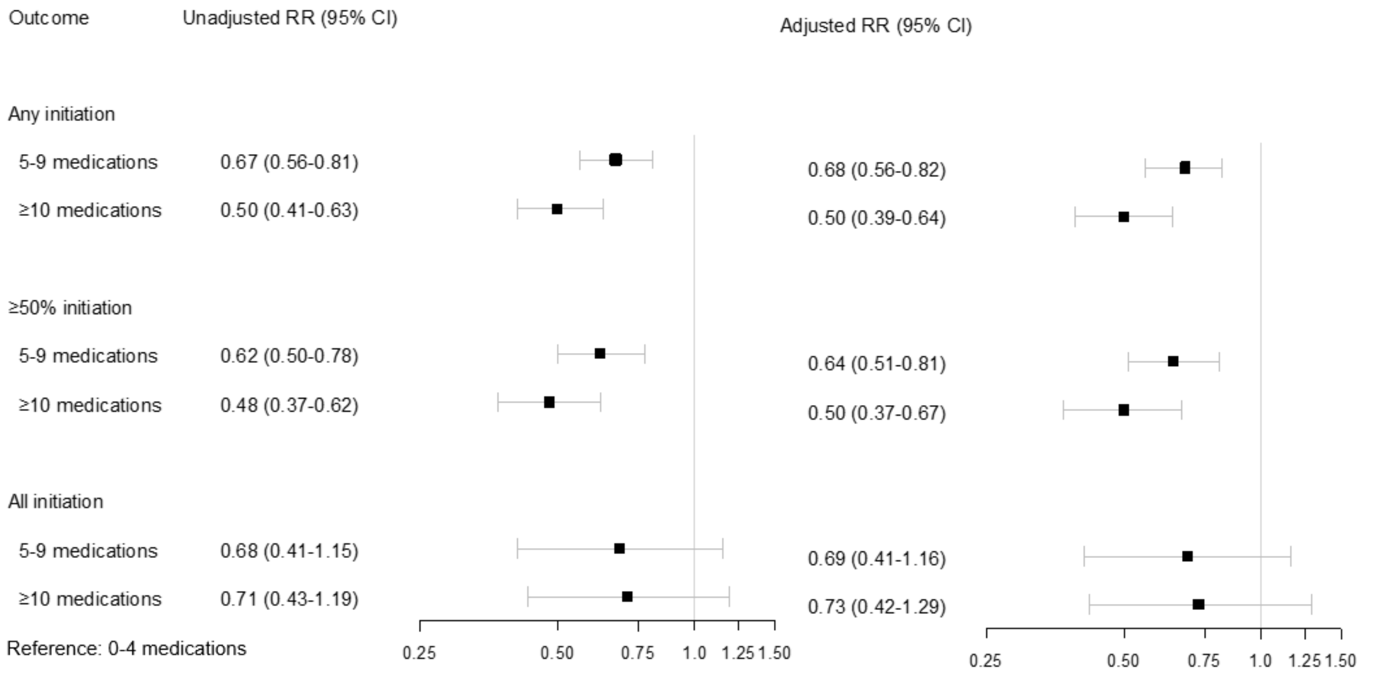

Supplement: Supplemental Data [file mmc1.pdf]
